# The Association of Ideal Cardiovascular Health and Atherogenic Index of Plasma in Rural Population: A Cross-Sectional Study from Northeast China

**DOI:** 10.3390/ijerph13101027

**Published:** 2016-10-19

**Authors:** Ye Chang, Yuan Li, Xiaofan Guo, Dongxue Dai, Yingxian Sun

**Affiliations:** Department of Cardiology, the First Hospital of China Medical University, Shenyang 110001, China; chang.ye@stu.xjtu.edu.cn (Y.C.); xi.aohan1989@163.com (Y.L.); guoxiaofan1986@foxmail.com (X.G.); 18240409506@163.com (D.D.)

**Keywords:** ideal cardiovascular health, atherogenic index of plasma, atherosclerosis, rural population

## Abstract

In 2010, the American Heart Association has proposed a new concept “ideal cardiovascular health” (CVH) based on seven CVH metrics: smoking, body mass index, physical activity, diet score, total cholesterol, blood pressure, and fasting plasma glucose. We aimed to determine the association of CVH with atherogenic index of plasma (AIP), a strong marker for atherosclerosis (AS). This cross-sectional study was conducted in the rural areas of northeast China and 11,113 middle-aged subjects were enrolled. Seven CVH metrics were classified into ideal, intermediate, and poor groups. AIP was calculated as log (TG/HDL) (triglycerides/high-density lipoprotein cholesterol). AIP > 0.21 was classified into the high AIP group and served as dependent variable. All seven CVH metrics were correlated with AIP. A gradient relationship between the number of poor CVH metrics and the prevalence of high AIP existed. Log binomial regression analysis showed that compared to those with five to seven ideal CVH metrics, individuals with four, three, two, one, and no ideal CVH metrics had 1.67, 2.66, 4.00, 5.30 and 6.50 times higher prevalence for high AIP. The subjects with poor CVH status had 2.73 times higher prevalence for high AIP. We found an inversely gradient relationship between the number of ideal CVH metrics and lower prevalence of high AIP.

## 1. Introduction

Cardiovascular diseases (CVD) have been the leading causes of morbidity and mortality worldwide according to the report from the American Heart Association (AHA) [1]. It is worth noting that the mortality rate from CVD has declined impressively in developed and high-income countries in the last several decades [2,3,4]. However, this is still of particular concern in developing and low/middle-income countries [5,6]. With the development of the economy, improvement in living and medical conditions, and extension of life expectancy in recent decades after the Reform and Opening-up policy, China gradually steps into aging society. Concomitantly, in China, CVD mortality rates increase over time and now have ranked first as a cause of death. Yang G. et al. reported that CVD accounted for approximately 32% of all causes of death in 2005 in China [7]. In 2014, Report on Cardiovascular Diseases in China was proposed by National Center for Cardiovascular Diseases (NCCD) in China [8] (or see the attachment). The report indicates that in China, the prevalence of CVD is continuously increasing and one in five Chinese adults (≈290 million) is afflicted by CVD. The report also indicates that the mortality rate due to CVD has been the leading cause of death and 44.8% of deaths in rural area and 41.9% of deaths in urban area are caused by CVD in China.

In 2010, the AHA proposed a new concept “ideal cardiovascular health (CVH)” based on seven CVH metrics: smoking, body mass index (BMI), physical activity, diet score, total cholesterol (TC), blood pressure (BP), and fasting plasma glucose (FPG) [9]. The AHA set the goal “by 2020, to improve the cardiovascular health of all Americans by 20% while reducing deaths from cardiovascular diseases and stroke by 20%” [9]. Although ideal CVH has shown protective role against CVD [10,11], abundant epidemiological studies worldwide demonstrated that the prevalence of ideal CVH was surprisingly low, ranging from 0.1%–3.3%, as detailed in a summary in our previous study [12]. Consistently, our previous study showed extremely low (0.1%) prevalence of ideal CVH in the rural population of northeast China [12]. Therefore, knowledge of CVH status and its association with CVD was essential for CVH promotion and disease prevention.

Atherosclerosis (AS) is the basic pathological mechanism of CVD and can lead to stroke and acute coronary syndrome. There are several subclinical AS markers, including carotid intima-media thickness (IMT) [13] and aortic IMT [14] detected by B-mode ultrasound, and coronary artery calcification (CAC) [15] detected by computed tomography (CT). Besides, dyslipidemia has been identified as major risk factor for AS [16]. Dyslipidemia describes as disorders of the lipid metabolism including four traditional pro-atherogenic lipid profile, high TC, low high-density lipoprotein cholesterol (HDL), elevated triglycerides (TG), and high low-density lipoprotein cholesterol (LDL) levels according to the ATP III criteria [17]. Compared to the traditional pro-atherogenic lipid profile, studies found that atherogenic index of plasma (AIP) was a significant predictor of AS and better than LDL [18,19]. AIP was calculated as log (TG/HDL) [18]. After the logarithmical transformation, AIP could correct for the lack of normative distribution and demonstrate a correlation with smaller LDL particles and increased fractional esterification rate (FER_HDL_) [18]. Besides AS, studies demonstrated that AIP was also significantly correlated with acute coronary events [20], CVD, and its risk factors [18,19,21,22].

Shen et al. indicated that the ideal CVH score correlated significantly with AIP, and a 1-point increase in the CVH score could lead to a 0.046 reduction in AIP and a 22.3% reduction in the high risk of developing AS (accessed by AIP > 0.21) in middle-aged men from southeastern China [23]. However, uncertainty remains regarding the relationship between the ideal CVH metrics and AIP because of the limited number of previous studies and lack of study in the general population. The present study was aimed to determine the association between ideal CVH and AIP in a general population from the rural areas of northeast China. This may be useful for prevention and control strategies for CVD.

## 2. Materials and Methods

### 2.1. Study Population

From January 2012 to August 2013, a representative sample of individuals aged ≥35 years was selected to present the prevalence, incidence and natural history of cardiovascular risk factors in rural areas of Liaoning Province, which was called Northeast China Rural Cardiovascular Health Study (NCRCHS). The study adopted a multi-stage, stratified, random cluster-sampling scheme. In the first stage, three counties (Dawa, Zhangwu, and Liaoyang County) were selected randomly from rural areas of Liaoning province. In the second stage, one town was randomly selected from each of the three counties. In the third stage, 26 rural villages from the three towns were randomly selected. Participants who were pregnant or had a malignant tumor or mental disorder were excluded from the present study. A total of 14,016 eligible permanent residents aged ≥35 years from each village were invited to participate in the study and 11,956 participants (i.e., response rate of 85.3%) agreed to participate and completed the present study. Only participants with a complete set of data regarding the variables analyzed in the present study were included, for a final sample size of 11,113 (5129 males and 5984 females).

Written consent was obtained from all participants after they had been informed of the objectives, benefits, medical items, and confidentiality of personal information. If the participants were illiterate, written informed consent was obtained from their proxies.

### 2.2. Ethical Statement

The study protocol was approved by the Ethics Committee of China Medical University (Shenyang, China, ethical approved project identification code: 2011-2-2), and all procedures were performed in accordance with good ethical standards.

### 2.3. Data Collection

Our survey was performed by cardiologists and trained nurses during a single visit at a clinic in each village. Before the survey was performed, all eligible investigators underwent training including study purpose and procedures, how to administer the questionnaire, standard methods of measurement, and importance of standardization. Only those who scored perfectly on a strict test at the end of training were allowed to become investigators. During data collection, study inspectors offered further instruction and support as needed.

The present study used a standardized questionnaire to collect data during a face-to-face interview on demographic characteristics, lifestyle risk factors, dietary habits, family income, and other variables. Their marital status was categorized into two groups: married or living with partner; and unmarried, divorced, or widowed. Ethnicity was classified as Han or others (including ethnic minorities in China, such as Mongol and Manchu). Family income was classified as 5000 or less; 5000–20,000; and 20,000 or over, CNY/year. Educational level was categorized as low (no schooling, incomplete primary education, and primary education), middle (three or four years of secondary education), and high (college and university education). Study participants were asked whether or not they currently drank alcohol (two or more times/week for at least one year).

### 2.4. Smoking Status

Study participants were asked the following questions: (1) whether or not have you been a smoker? If yes, then; (2) are you a smoker at present? (3) have you ever quitted smoking? If yes, then; (4) how long have you quit smoking? 1 = less than 6 months, 2 = more than 6 months and less than 1 year, 3 = more than 1 year and less than 2 years, 4 = more than 2 years. Then smoking was classified as ideal (never smoker or having quit over 12 months before), intermediate (having quit 12 months or less before), or poor (current smoker).

### 2.5. Diet Score

The questionnaire included items related to average consumption (grams per week) of several food items (including legumes, vegetables, fruits, fish, poultry, and salt intake). Healthy diet was originally defined using the following five components: (1) legumes and cereals as basic food; (2) 500 g or over fruits and vegetables daily; (3) less than 100 g red meat/day; (4) regular (in most weeks) intake of soybean products and/or unprocessed fish; and (5) preference for non-salty food, in accordance with the current “Dietary Guidelines for Chinese Residents” [24]. Diet score was classified as ideal (4–5 components), intermediate (2–3 components), or poor (0–1 components).

### 2.6. Physical Activity

As for physical activity, we initially set questions as “Do you regularly exercise?”, if yes, “how many times do you exercise per week and how long do you exercise every time?” and “what is your most commonly used way of exercise? 1 = walking, 2 = running, 3 = swimming, 4 = ball games, 5 = mountaineering, 6 = others”. During the visit, we found that these questions did not apply to the rural populations. Most rural populations did not actively engage in exercise such as walking, running, swimming, ball games, or mountaineering. They preferred to watch TV or play mahjong or poker in their leisure time (these are not conclusions from our questionnaire), as specially indicated in our previous study [12]. Then we decided to adopt another method, described elsewhere, to measure occupational physical activity [25]. Briefly, participants were asked: “which type do you think your occupational physical activity belongs to?” Occupational physical activity was grouped into three categories: (1) low was defined as participants who reported light levels of occupational physical activity, such as the elderly, crippled, and paralyzed; (2) moderate was defined as participants who reported moderate occupational physical activity, such as drivers and office workers; (3) high was defined as participants who reported high level of occupational physical activity, such as manual agricultural activities and miner. To assist with the AHA’s criterion [9], we transformed low, moderate, and high to poor, intermediate, and ideal in our modified criteria.

### 2.7. Category of Blood Pressure (BP)

According to AHA protocol [26], BP was measured three times at two minute intervals after at least five minutes of rest using a standardized automatic electronic sphygmomanometer (HEM-907; Omron, Kyoto, Japan). The study participants were advised to avoid caffeinated beverages and exercise for at least 30 min before the measurement. During the measurement, the participants were seated with the arm supported at the level of the heart. The mean of three BP measurements was calculated and used in all analyses. According to the AHA definition [9], BP was classified as ideal (systolic blood pressure (SBP) < 120 mmHg and diastolic blood pressure (DBP) < 80 mmHg, untreated), intermediate (SBP 120–139 mmHg or DBP 80–89 mmHg, or treated to goal), and poor (SBP ≥ 140 mmHg or DBP ≥ 90 mmHg).

### 2.8. Category of BMI

Weight and height was measured to the nearest 0.1 kg and 0.1 cm, respectively with the participants in light-weight clothing and without shoes. Waist circumference (WC) was measured at the umbilicus using a non-elastic tape (to the nearest 0.1 cm), with the participants standing at the end of normal expiration. BMI was calculated as weight in kilograms divided by the square of the height in meters. BMI was categorized into three groups as normal (BMI < 22.9 kg/m^2^), overweight (23 ≤ BMI < 27.4 kg/m^2^), and obese (BMI ≥ 27.5 kg/m^2^), according to the World Health Organization (WHO) obesity criteria for Asian people [27].

### 2.9. Serum Analysis

Fasting blood samples were collected in the morning after at least 12 h of fasting for all participants. Blood samples were obtained from an antecubital vein into vacutainer tubes containing EDTA. FPG, TC and other routine blood biochemical indexes were analyzed enzymatically on an autoanalyzer (SYSMEX, Kobe, Japan). All laboratory equipment was calibrated, and blinded duplicate samples were used. TC was classified as ideal (<200 mg/dL (5.18 mmol/L), untreated), intermediate (200–239 mg/dL (5.18–6.21 mmol/L) or drug treated to goal), or poor (≥240 mg/dL (6.21 mmol/L)). FPG was classified as ideal (<100 mg/dL (5.6 mmol/L), untreated), intermediate (100–125 mg/dL (5.6–7.0 mmol/L) or drug treated to goal), or poor (≥126 mg/dL (7.0 mmol/L)).

### 2.10. Definition of AIP and Ideal CVH

According to previous studies, AIP was calculated as log (TG/HDL) and was classified into three groups: low- (<0.11), intermediate- (0.11–0.21) and high-risk (>0.21) [18,23,28,29]. In accordance with definition by previous studies [9,30], ideal health is all seven health metrics at ideal levels, intermediate health is at least one health metric at intermediate level, but no poor health metrics, and poor health is at least one of seven health metrics at poor level. Comparison of the AHA’s criteria and the criteria in present study on the seven metrics of CVH was shown in detail in our previous study [12].

### 2.11. Statistical Analyses

Descriptive statistics were calculated for all the variables, including continuous variables (reported as mean ± SD) and categorical variables (reported as numbers and percentages). Differences in the subgroups were compared using Student’s *t*-test for continuous variables. Comparisons of categorical variables between groups were performed using the χ^2^ test. Log binomial regression (generalized linear model with a logarithmic link function and binomial distribution for the residual) was used to identify association between CVH metrics and the prevalence of high AIP, with adjustment for age, sex, race, education, family income, and drinking. Analyses were presented as prevalence ratio (PR) and 95% confidence interval (95% CI). All the statistical analyses were performed using SPSS version 22.0 software (SPSS Inc., Chicago, IL, USA), and all statistical tests were two-sided with the significance level set at *p* ≤ 0.05.

## 3. Results

A total of 11,113 subjects (5129 males and 5984 females) aged ≥35 years participated in the study. Overall, there were 2572 subjects with a high AIP, accounting for 23.1% of our study population.

Table 1 showed the clinical and demographic characteristics of study population according to AIP (low-intermediate vs. high). The average age in our study was 53.8 ± 10.6 years and subjects at high AIP group were older. No sex or race differences existed in the two groups. Overall, the education level and family income were low in the rural areas of northeast China and showed no differences in both groups. Subjects with a high AIP had a lower drinking rate. In addition, the values of BMI and WC were larger in high AIP group than that in low-intermediate AIP group. Furthermore, the values of SBP, DBP, FPG, TC, TG, LDL, and AIP were significantly higher in high AIP group compared with the low-intermediate AIP group, while the value of HDL showed the opposite trend.

**Figure 1:**
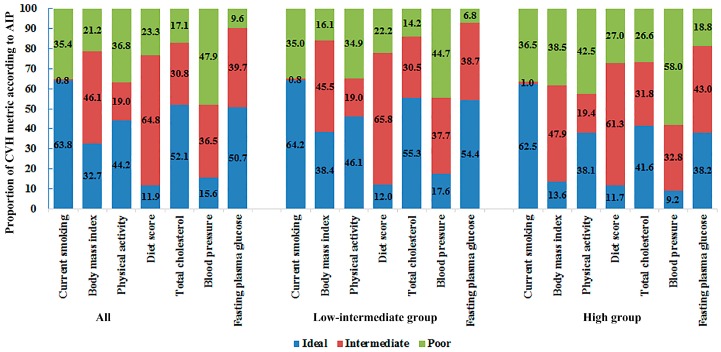
The distribution of CVH metrics according to AIP (low-intermediate vs. high). AIP, atherogenic index of plasma; CVH, cardiovascular health.

Figure 1 showed the distribution of CVH metrics according to AIP (low-intermediate vs. high). The current smoking status showed no differences in the two groups. Overall, the percentages of ideal CVH metrics were lower in high AIP group and the percentages of poor CVH metrics were higher in the low-intermediate AIP group. The percentages of ideal CVH metrics in two groups (low-intermediate vs. high) were as follows: 38.4% vs. 13.6% for BMI; 46.1% vs. 38.1% for physical activity; 12.0% vs. 11.7% for diet score; 55.3% vs. 41.6% for TC; 17.6% vs. 9.2% for BP; and 54.4% vs. 38.2% for FPG (all *p*-values < 0.001). The percentages of poor CVH metrics in two groups (low-intermediate vs. high) were as follows: 16.1% vs. 38.5% for BMI; 34.9% vs. 42.6% for physical activity; 22.2% vs. 27.1% for diet score; 14.2% vs. 26.6% for TC; 44.8% vs. 58.0% for BP; and 6.9% vs. 18.8% for FPG.

Table 2 showed the results of multiple linear regression on the association between each CVH metric and AIP. All seven CVH metrics were independently related to AIP, with adjustment for age, sex, race, spouse, education, family income, and drinking.

Table 3 showed the PRs and 95% CI of having a high AIP based on each CVH metric. High AIP group served as a dependent variable, and the seven CVH metrics served as independent variables. Then these variables were included in the log binomial regression model with adjustment for age, sex, race, spouse, education, family income, and drinking. Compared to subjects in ideal CVH metric group, participants in poor CVH metric group had remarkable higher prevalence for high AIP. The prevalence of high AIP was positively associated with current smoking (PR: 1.26, 95% CI: 1.13–1.40), poor BMI (PR: 3.76, 95% CI: 3.27–4.32), poor physical activity (PR: 1.21, 95% CI: 1.09–1.35), poor diet score (PR: 1.30, 95% CI: 1.11–1.53), poor TC (PR: 1.56, 95% CI: 1.39–1.75), poor BP (PR: 1.31, 95% CI: 1.11–1.54), and poor FPG (PR: 1.90, 95% CI: 1.66–2.18).

The prevalence of high AIP showed a graded relation to the number of ideal CVH metrics. As shown in Table 4, none had high AIP among the subjects who met all seven ideal health metrics. The prevalence of high AIP were very low among subjects with five to seven ideal CVH metrics (0%, 8.7%, and 7.7%, respectively). While the prevalence of high AIP increased as the number of ideal CVH metrics decreased, reaching 44.6% among subjects without ideal CVH metrics. Because only 14 subjects had seven ideal CVH metrics, and none had high AIP among them, we could not take them as reference when running the log binomial regression. Therefore, we classified the subjects with five to seven ideal CVH metrics into one group and took them as reference. After adjustment for age, sex, race, spouse, education, family income, and drinking, the PR of having a high AIP correspondingly increased with decreasing numbers of ideal CVH metrics. Subjects with four, three, two, one, and no ideal CVH metrics had 1.67 (95% CI: 1.28–2.17), 2.66 (95% CI: 2.08–3.40), 4.00 (95% CI: 3.14–5.09), 5.30 (95% CI: 4.13–6.79), and 6.50 (95% CI: 4.79–8.83) times higher prevalence for high AIP compared with subjects who met five to seven ideal health metrics.

As shown in Table 5, according to the categories of ideal CVH defined by AHA, the prevalence of high AIP was 0% among subjects with ideal CVH, 9.4% among subjects with intermediate CVH and 25.4% among subjects with poor CVH. None had high AIP among them, we could not take them as reference when running the log binomial regression. Therefore, we took ideal and intermediate CVH as reference, subjects with poor CVH thus had 2.73 (95% CI: 2.25–3.31) times higher prevalence for high AIP, after adjustment for age, sex, race, spouse, education, family income, and drinking.

## 4. Discussion

In this cross-sectional study, we estimated the association between ideal CVH and AIP in general population from the rural areas of northeast China. High AIP was defined as AIP > 0.21 and served as dependent variable. We mainly found that the percentages of ideal CVH metrics were lower in the high AIP group, and subjects with poor CVH metrics had higher prevalence for high AIP. Furthermore, an inverse gradient relationship existed between the number of ideal CVH metrics and the prevalence of high AIP. Compared to those with five to seven ideal CVH metrics, individuals with four, three, two, one, and no ideal CVH metrics had 1.67, 2.66, 4.00, 5.30, and 6.50 times higher prevalence for high AIP. Individuals with poor CVH had 2.73 times higher prevalence for high AIP compared to those with ideal CVH.

There are several subclinical AS markers, including carotid intima-media thickness (IMT) [13] and aortic IMT [14] detected by B-mode ultrasound, and coronary artery calcification (CAC) detected by computed tomography (CT) [15]. Several studies have accessed the relationship between CVH and AS markers [13,23,31]. Kulshreshtha A. et al. confirmed that ideal CVH had a strong inverse correlation with carotid IMT, another preclinical marker for AS [13]. In a STRIP study conducted in adolescents, Pahkala K. et al. found that the number of ideal CVH metrics was inversely associated with aortic IMT and directly associated with elasticity [14]. Intracranial artery stenosis (ICAS) is one of the most common causes of ischemic stroke in Asia and its basic pathological mechanism is AS. Zhang Q. et al. demonstrated that clear gradient relationship between the number of ideal CVH metrics and lower prevalence of ICAS in a Chinese population [31]. CAC is another established marker of subclinical AS. Saleem Y. et al. found that those with a favorable ideal CVH score had a lower prevalence and severity of subclinical AS estimated by CAC score [32]. Consistently, in northern Chinese population, Luo T.Y. et al. found that the participants with more ideal CVH metrics had a lower prevalence of subclinical AS estimated by CAC score [15]. However, in large-scale epidemiological investigations, it is too inconvenient to use B-mode ultrasound or CT to access AS.

AIP was another significant predictor of AS [18,19] and was more convenient and economical compared B-mode ultrasound or CT. Shen et al. indicated that the ideal CVH score correlated significantly with AIP, and a 1-point increase in the CVH score could lead to a 0.046 reduction in AIP and a 22.3% reduction in the high risk of developing AS [23]. The study subjects in the study conducted by Shen et al. were middle-aged men in southeastern China. Uncertainty remains regarding the relationship between the ideal CVH metrics and AIP because of limited studies on general populations. Considering the information indicating the potentially unique utility of AIP as an independent marker of CVD risk, and the relatively small body of research examining AIP as an outcome variable, the present study was also designed to explore this lipid ratio as the primary outcome of interest. We found all of the seven CVH metrics were significantly correlated with AIP. BMI exhibited the greatest effect on AIP (PR: 3.76, 95% CI: 3.27–4.32), followed by the FPG level, TC level, BP level, diet score, smoking status, and physical activity, after adjustment for covariates. Epidemiological studies have shown associations of BMI, FPG, TC, diet, BP, physical activity, and smoking with AIP (either increased TG or decreased HDL) [22,23,29,33,34]. An inverse gradient relationship existed between the number of ideal CVH metrics and the prevalence of high AIP. Consistent with previous study [23], our findings indicated that the subjects with poor CVH had higher prevalence for high AIP and were prone to suffering AS. Therefore, health education and promotion and lifestyle interventions in high-risk populations with poor CVH will result in a reduction in the prevalence of AS and CVD in general population. Notably, poor lifestyles should be improved among middle-aged populations with overweight/obesity, smoking, poor status of diet score, and physical activity based on the seven CVH metrics. In addition, the levels of BP, FPG, and blood lipid should be monitored periodically and timely adjustment of medications is suggested.

We also found some differences between our study and previous studies. (1) Age made a big difference. In adolescents, the effect of the score on aortic IMT nearly plateaued after four ideal metrics; for elasticity, a similar phenomenon was seen after three of the metrics were met [14]. However, in adults, a somewhat more linear, graded association between the ideal CVH scores with carotid IMT [13], ICAS [31], and AIP [23]. In adolescence, one does not have to reach all seven metrics to gain CVH benefits; the key is to avoid having a low ideal CVH score [14]. In adulthood, one still can gain CVH benefits even the ideal CVH metrics are more than five or six. In the AGES-Reykjavik study [35], the average age was 75.6 ± 5.1 years. Sturlaugsdottir R. et al. [35] reported that carotid IMT and total plaque area (TPA) progression over 5 years were independent of the CVH scores. (2) Study sample ranged from hundreds to tens of thousands subjects. Therefore, a selection bias for survivors may existed in these studies. (3) Different CVH metrics had various effects in different studies. We found that BMI exhibited the greatest effect on AIP (PR: 3.76, 95% CI: 3.27–4.32), the remaining CVH metrics did not show significant differences. However, Kulshreshtha et al. [13] found that the overall association of CVH and carotid IMT was primarily driven by health factors—such as BP, FPG, and TC—instead of health behaviors. Zhang Q. et al. [31] found that, besides smoking, the remaining health behaviors (BMI, diet, and physical activity) showed no significant relationship with the prevalence of ICAS, while all of the health factors (smoking, TC, BP, and FPG) were significantly associated with the prevalence of ICAS, after adjustment for sex, age, education, income, and family history of stroke.

We also noticed that although the criteria for ideal CVH were based on the AHA criteria, there were minor adjustments for BMI (WHO criterion or Asia criterion) and diet score (or salt intake per day) in different studies. Besides, the ideal criterion for physical activity are quite different in studies. In the Heart SCORE study, physical activity was evaluated using the Lipid Research Clinic questionnaire and the authors indicated that although the amount of exercise (min/week) could not be derived, the questionnaire provided approximations of ideal, intermediate, and poor physical activity [36]. In one twin study, physical activity was determined by means of a modified version of the Baecke Questionnaire of habitual physical activity and the cumulative Baecke score were used to classify individuals into poor, intermediate, and ideal levels of physical activity [13]. There are many other criteria for physical activity in different studies. Furthermore, observational and experimental studies have shown that regular practice of physical activity could induce desirable changes in plasma lipid levels [37], especially HDL increase and TG decrease [38]. Study found that the reduction in TG levels was associated with higher physical activity intensity, but not with physical activity frequency [37]. Another study reinforced the benefits of physical activity on HDL and TG levels, with a slight advantage for vigorous physical activity as compared to physical activity duration of 150 min/week [39]. In our study, occupational physical activity intensity was used to access the status of physical activity and could provide approximations of ideal, intermediate, and poor physical activity recommended by the AHA. In short, all these factors including age, sex, race, sample size, and modified criteria for ideal CVH may contribute to inconsistencies in different studies.

## 5. Limitations

Some limitations should also be considered in light of these results. Firstly, this was a cross-sectional study and could not definitively provide causal relationship between the health factors/behaviors and the prevalence of high AIP. Second, participants enrolled in our study were from rural areas of China, thus, the results should be interpreted cautiously. Third, some criteria for ideal CVH in this study were adjusted such as the criteria for BMI, physical activity, and diet score, which may make it difficult to compare with other studies.

## 6. Conclusions

In summary, our study showed that the CVH metrics were strongly correlated with AIP in general population from the rural area of northeast China. The association of CVH metrics and AIP were driven by both health behaviors and factors. Furthermore, we observed a clear gradient inverse relationship between the number of ideal CVH metrics and the prevalence of high AIP. Ideal CVH metrics were significant for the prevention and control of AS.

## Figures and Tables

**Table 1:** Baseline characteristics of study population.

Variables	Total (*n* = 11,113)	Low-Intermediate AIP (*n* = 8541)	High AIP (*n* = 2572)	*p*-Value
Age (year)	53.8 ± 10.6	53.8 ± 10.7	54.4 ± 10.2	<0.01
Male (%)	5129 (46.2)	3909 (45.8)	1220 (47.4)	0.137
Spouse (live, %)	10,261 (91.6)	7814 (91.5)	2369 (92.1)	0.320
Education (%)				0.325
Primary school or below	5528 (49.7)	4192 (49.1)	1336 (51.9)	
Middle school	4539 (40.8)	3573 (41.8)	996 (37.6)	
High school or above	1046 (9.4)	776 (9.1)	270 (10.5)	
Family income (CNY/year, %)				0.248
≤5000	1375 (12.4)	1047 (12.3)	328 (12.8)	
5000–20,000	6060 (54.5)	4710 (55.1)	1350 (52.5)	
>20,000	3678 (33.1)	2784 (32.6)	894 (34.7)	
Race				0.20
Han	10,540 (94.8)	8088 (94.7)	2452 (95.3)	
Others ^a^	573 (5.2)	453 (5.3)	120 (4.7)	
Current smoking status (%)	3932 (35.4)	2994 (35.1)	938 (36.5)	0.190
Current drinking status (%)	2502 (22.5)	1969 (23.1)	533 (20.7)	<0.05
Diet score	2.33 ± 1.13	2.35 ± 1.11	2.26 ± 1.17	<0.001
SBP (mmHg)	141.6 ± 23.4	140.2 ± 23.3	146.3 ± 23.3	<0.001
DBP (mmHg)	82.0 ± 11.8	81.1 ± 11.6	85.1 ± 11.9	<0.001
BMI (kg/m^2^)	24.8 ± 3.7	24.2 ± 3.5	26.6 ± 3.5	<0.001
WC (cm)	82.4 ± 9.8	81.8 ± 9.5	87.9 ± 8.9	<0.001
FPG (mmol/L)	5.90 ± 1.64	5.75 ± 1.38	6.42 ± 2.22	<0.001
TC (mmol/L)	5.24 ± 1.09	5.13 ± 1.01	5.57 ± 1.26	<0.001
TG (mmol/L)	1.64 ± 1.51	1.13 ± 0.44	3.32 ± 2.34	<0.001
LDL-C (mmol/L)	2.93 ± 0.82	2.87 ± 0.79	3.10 ± 0.91	<0.001
HDL-C (mmol/L)	1.41 ± 0.38	1.49 ± 0.38	1.12 ± 0.22	<0.001
AIP	−0.089 ± 0.315	−0.140 ± 0.210	0.425 ± 0.194	<0.001

Data are expressed as the mean (SD) or as *n* (%). Abbreviations: AIP: atherogenic index of plasma; BMI: body mass index; CNY: China Yuan; DBP: diastolic blood pressure; FPG: fasting plasma glucose; HDL-C: high-density lipoprotein cholesterol; LDL-C: low-density lipoprotein cholesterol; SBP: systolic blood pressure; TC: total cholesterol; TG: triglycerides; WC: waist circumference; ^a^ Including some ethnic minorities in China, such as Mongol and Manchu.

**Table 2:** Multiple linear regression on the association between each CVH metric and AIP ^a^.

Metrics	β	*p*
Current smoking	0.083	<0.001
Body mass index	0.305	<0.001
Physical activity	0.067	<0.001
Diet score	−0.045	<0.001
Total cholesterol	0.148	<0.001
Systolic blood pressure	−0.108	<0.001
Diastolic blood pressure	0.140	<0.001
Fasting plasma glucose	0.134	<0.001

^a^ Adjustment for age, sex, race, education, family income, and drinking. β = standardized regression coefficient.

**Table 3:** Log binomial regression on the association between each CVH metric and having a high AIP ^a^.

Metrics	Prevalence Ratio	95% CI	*p*-Value
Current smoking			
Ideal	1.00		
Intermediate	1.24	0.79–2.00	0.346
Poor	1.26	1.13–1.40	<0.001
Body mass index			
Ideal	1.00		
Intermediate	2.33	2.05–2.65	<0.001
Poor	3.76	3.27–4.32	<0.001
Physical activity			
Ideal	1.00		
Intermediate	1.14	1.01–1.29	<0.05
Poor	1.21	1.09–1.35	<0.001
Diet score			
Ideal	1.00		
Intermediate	1.00	0.87–1.15	0.999
Poor	1.30	1.11–1.53	<0.001
Total cholesterol			
Ideal	1.00		
Intermediate	1.15	1.03–1.27	<0.05
Poor	1.56	1.39–1.75	<0.001
Blood pressure			
Ideal	1.00		
Intermediate	1.22	1.04–1.43	<0.05
Poor	1.31	1.12–1.54	<0.001
Fasting plasma glucose			
Ideal	1.00		
Intermediate	1.25	1.13–1.38	<0.001
Poor	1.90	1.66–2.18	<0.001

^a^ Adjustment for age, sex, race, education, family income, and drinking.

**Table 4:** Prevalence and prevalence ratios of having a high AIP according to number of ideal CVH metrics ^a^.

Number of Ideal CVH Metrics	Total Number (%) in Category	Number at High AIP Group (% Having a High AIP in Category)	Prevalence Ratio (95% CI)
7	14 (0.1)	0 (0)	(5–7 ideal CVH metrics)
6	208 (1.9)	18 (8.7)	
5	816 (7.3)	63 (7.7)	
4	1996 (18.0)	250 (12.5)	1.67 (1.28, 2.17)
3	2982 (26.8)	578 (19.4)	2.66 (2.08, 3.40)
2	2989 (26.9)	853 (28.5)	4.00 (3.14, 5.09)
1	1785 (16.1)	666 (37.3)	5.30 (4.13, 6.79)
0	323 (2.9)	144 (44.6)	6.50 (4.79, 8.83)

^a^ Adjustment for age, sex, race, education, family income, and drinking.

**Table 5:** Prevalence and prevalence ratios of having a high AIP according to CVH categories ^a^.

Categories of CVH Metrics	Total Number (%) in Category	Number at High AIP Group (% Having a High AIP in Category)	Prevalence Ratio (95% CI)
Ideal	14 (0.1)	0 (0)	(ideal + intermediate)
Intermediate	1300 (11.7)	122 (9.4)	
Poor	9799 (88.2)	2450 (25.0)	2.73 (2.25, 3.31)

^a^ Adjustment for age, sex, race, education, family income, and drinking.

## References

[1] Lloyd-Jones D., Adams R.J., Brown T.M., Carnethon M., Dai S., De Simone G., Ferguson T.B., Ford E., Furie K., Gillespie C. (2010). Heart disease and stroke statistics—2010 update: A report from the American Heart Association. Circulation.

[2] Capewell S., Ford E.S., Croft J.B., Critchley J.A., Greenlund K.J., Labarthe D.R. (2010). Cardiovascular risk factor trends and potential for reducing coronary heart disease mortality in the United States of America. Bull. WHO.

[3] Ford E.S., Capewell S. (2011). Proportion of the decline in cardiovascular mortality disease due to prevention versus treatment: Public health versus clinical care. Annu. Rev. Public Health.

[4] Ford E.S., Ajani U.A., Croft J.B., Critchley J.A., Labarthe D.R., Kottke T.E., Giles W.H., Capewell S. (2007). Explaining the decrease in U.S. deaths from coronary disease, 1980–2000. N. Engl. J. Med..

[5] Fuster V., Kelly B.B., IOM (Institute of Medicine) (2010). Promoting Cardiovascular Health in the Developing World: A Critical Challenge to Achieve Global Health.

[6] Jankovic S., Stojisavljevic D., Jankovic J., Eric M., Marinkovic J. (2014). Status of cardiovascular health in a transition European country: Findings from a population-based cross-sectional study. Int. J. Public Health.

[7] Yang G., Kong L., Zhao W., Wan X., Zhai Y., Chen L.C., Koplan J.P. (2008). Emergence of chronic non-communicable diseases in China. Lancet.

[8] National Center for Cardiovascular Diseases, China (2015). Report on Cardiovascular Diseases in China (2016).

[9] Lloyd-Jones D.M., Hong Y., Labarthe D., Mozaffarian D., Appel L.J., Van Horn L., Greenlund K., Daniels S., Nichol G., Tomaselli G.F. (2010). Defining and setting national goals for cardiovascular health promotion and disease reduction: The American Heart Association’s strategic impact goal through 2020 and beyond. Circulation.

[10] Dong C., Rundek T., Wright C.B., Anwar Z., Elkind M.S., Sacco R.L. (2012). Ideal cardiovascular health predicts lower risks of myocardial infarction, stroke, and vascular death across whites, blacks, and hispanics: The Northern Manhattan Study. Circulation.

[11] Zhang Q., Zhou Y., Gao X., Wang C., Zhang S., Wang A., Li N., Bian L., Wu J., Jia Q. (2013). Ideal cardiovascular health metrics and the risks of ischemic and intracerebral hemorrhagic stroke. Stroke.

[12] Chang Y., Guo X., Chen Y., Guo L., Li Z., Yu S., Yang H., Sun G., Sun Y. (2016). Prevalence and metrics distribution of ideal cardiovascular health: A population-based, cross-sectional study in rural China. Heart Lung Circ..

[13] Kulshreshtha A., Goyal A., Veledar E., McClellan W., Judd S., Eufinger S.C., Bremner J.D., Goldberg J., Vaccarino V. (2014). Association between ideal cardiovascular health and carotid intima-media thickness: A twin study. J. Am. Heart Assoc..

[14] Pahkala K., Hietalampi H., Laitinen T.T., Viikari J.S.A., Ronnemaa T., Niinikoski H., Lagstrom H., Talvia S., Jula A., Heinonen O.J. (2013). Ideal cardiovascular health in adolescence: Effect of lifestyle intervention and association with vascular intima-media thickness and elasticity (the STRIP study). Circulation.

[15] Luo T.Y., Liu X.H., Dai T.Y., Liu X.M., Zhang Q., Dong J.Z. (2016). Ideal cardiovascular health metrics and coronary artery calcification in northern Chinese population: A cross-sectional study. Biomed. Environ. Sci..

[16] Shen S.W., Lu Y., Li F., Shen Z.H., Xu M., Yao W.F., Feng Y.B., Yun J.T., Wang Y.P., Ling W. (2015). Potential long-term effects of previous schistosome infection may reduce the atherogenic index of plasma in Chinese men. Int. J. Parasitol..

[17] Expert Panel on Detection, Evaluation and Treatment of High Blood Cholesterol in Adults (2001). Executive summary of the third report of the national cholesterol education program (NCEP) expert panel on detection, evaluation, and treatment of high blood cholesterol in adults (adult treatment panel III). JAMA.

[18] Dobiasova M., Frohlich J. (2001). The plasma parameter log (TG/HDL-C) as an atherogenic index: Correlation with lipoprotein particle size and esterification rate in apob-lipoprotein-depleted plasma (FER_HDL_). Clin. Biochem..

[19] Dobiasova M., Frohlich J. (2000). The new atherogenic plasma index reflects the triglyceride and HDL-cholesterol ratio, the lipoprotein particle size and the cholesterol esterification rate: Changes during lipanor therapy. Vnitrni Lekarstvi.

[20] Dobiasova M., Urbanova Z., Samanek M. (2005). Relations between particle size of HDL and LDL lipoproteins and cholesterol esterification rate. Physiol. Res..

[21] Onat A., Can G., Kaya H., Hergenc G. (2010). “Atherogenic index of plasma” (log_10_ triglyceride/high-density lipoprotein-cholesterol) predicts high blood pressure, diabetes, and vascular events. J. Clin. Lipidol..

[22] Niroumand S., Khajedaluee M., Khadem-Rezaiyan M., Abrishami M., Juya M., Khodaee G., Dadgarmoghaddam M. (2015). Atherogenic index of plasma (AIP): A marker of cardiovascular disease. Med. J. Islam. Repub. Iran.

[23] Shen S., Lu Y., Qi H., Li F., Shen Z., Wu L., Yang C., Wang L., Shui K., Wang Y. (2016). Association between ideal cardiovascular health and the atherogenic index of plasma. Medicine.

[24] Ge K. (2011). The transition of Chinese dietary guidelines and food guide pagoda. Asia Pac. J. Clin. Nutr..

[25] Hu G., Tuomilehto J., Silventoinen K., Barengo N., Jousilahti P. (2004). Joint effects of physical activity, body mass index, waist circumference and waist-to-hip ratio with the risk of cardiovascular disease among middle-aged Finnish men and women. Eur. Heart J..

[26] Pickering T.G., Hall J.E., Appel L.J., Falkner B.E., Graves J., Hill M.N., Jones D.W., Kurtz T., Sheps S.G., Roccella E.J. (2005). Recommendations for blood pressure measurement in humans and experimental animals part 1: Blood pressure measurement in humans: A statement for professionals from the subcommittee of professional and public education of the American Heart Association Council on high blood pressure research. Circulation.

[27] Appropriate Body-Mass Index for Asian Populations and Its Implications for Policy and Intervention Strategies. http://www.who.int/nutrition/publications/bmi_asia_strategies.pdf.

[28] Raslova K., Dobiasova M., Hubacek J.A., Bencova D., Sivakova D., Dankova Z., Franekova J., Jabor A., Gasparovic J., Vohnout B. (2011). Association of metabolic and genetic factors with cholesterol esterification rate in HDL plasma and atherogenic index of plasma in a 40 years old Slovak population. Physiol. Res..

[29] Akbas E.M., Timuroglu A., Ozcicek A., Ozcicek F., Demirtas L., Gungor A., Akbas N. (2014). Association of uric acid, atherogenic index of plasma and albuminuria in diabetes mellitus. Int. J. Clin. Exp. Med..

[30] Folsom A.R., Yatsuya H., Nettleton J.A., Lutsey P.L., Cushman M., Rosamond W.D., Investigators A.S. (2011). Community prevalence of ideal cardiovascular health, by the American Heart Association definition, and relationship with cardiovascular disease incidence. J. Am. Coll. Cardiol..

[31] Zhang Q., Zhang S., Wang C., Gao X., Zhou Y., Zhou H., Wang A., Wu J., Bian L., Wu S. (2013). Ideal cardiovascular health metrics on the prevalence of asymptomatic intracranial artery stenosis: A cross-sectional study. PLoS ONE.

[32] Saleem Y., DeFina L.F., Radford N.B., Willis B.L., Barlow C.E., Gibbons L.W., Khera A. (2015). Association of a favorable cardiovascular health profile with the presence of coronary artery calcification. Circ. Cardiovasc. Imaging.

[33] Nansseu J.R., Moor V.J., Nouaga M.E., Zing-Awona B., Tchanana G., Ketcha A. (2016). Atherogenic index of plasma and risk of cardiovascular disease among Cameroonian postmenopausal women. Lipids Health Dis..

[34] Nunes S.O., Piccoli de Melo L.G., Pizzo de Castro M.R., Barbosa D.S., Vargas H.O., Berk M., Maes M. (2015). Atherogenic index of plasma and atherogenic coefficient are increased in major depression and bipolar disorder, especially when comorbid with tobacco use disorder. J. Affect. Disord..

[35] Sturlaugsdottir R., Aspelund T., Bjornsdottir G., Sigurdsson S., Eiriksdottir G., Imai C.M., Garcia M., Launer L.J., Harris T.B., Gudnason V. (2015). Carotid atherosclerosis and cardiovascular health metrics in old subjects from the AGES-Reykjavik study. Atherosclerosis.

[36] Bambs C., Kip K.E., Dinga A., Mulukutla S.R., Aiyer A.N., Reis S.E. (2011). Low prevalence of “ideal cardiovascular health” in a community-based population: The heart strategies concentrating on risk evaluation (heart score) study. Circulation.

[37] Halverstadt A., Phares D.A., Wilund K.R., Goldberg A.P., Hagberg J.M. (2007). Endurance exercise training raises high-density lipoprotein cholesterol and lowers small low-density lipoprotein and very low-density lipoprotein independent of body fat phenotypes in older men and women. Metabolism.

[38] Kraus W.E., Houmard J.A., Duscha B.D., Knetzger K.J., Wharton M.B., McCartney J.S., Bales C.W., Henes S., Samsa G.P., Otvos J.D. (2002). Effects of the amount and intensity of exercise on plasma lipoproteins. N. Engl. J. Med..

[39] Silva R.C., Diniz Mde F., Alvim S., Vidigal P.G., Fedeli L.M., Barreto S.M. (2016). Physical activity and lipid profile in the ELSA-Brasil study. Arq. Bras. Cardiol..

